# The anticancer effect of saffron in two p53 isogenic colorectal cancer cell lines

**DOI:** 10.1186/1472-6882-12-69

**Published:** 2012-05-28

**Authors:** Khuloud Bajbouj, Jan Schulze-Luehrmann, Stefanie Diermeier, Amr Amin, Regine Schneider-Stock

**Affiliations:** 1 Biology Department, Faculty of Science, UAE University, Al-Ain, United Arab Emirates; 2 Experimental Tumorpathology, Institute of Pathology, University of Erlangen-NÃ¼rnberg, UniversitÃ¤tsstrasse 22, Erlangen 91054, Germany; 3 Department of Zoology, Cairo University, Cairo, , Egypt

## Abstract

**Background:**

Saffron extract, a natural product, has been shown to induce apoptosis in several tumor cell lines. Nevertheless, the p53-dependency of saffron’s mechanism of action in colon cancer remains unexplored.

**Material and methods:**

In order to examine saffron’s anti-proliferative and pro-apoptotic effects in colorectal cancer cells, we treated two p53 isogenic HCT116 cell lines (HCT wildtype and HCT p53−/−) with different doses of the drug and analyzed cell proliferation and apoptosis in a time-dependent manner. MTT viability and crystal violet assays were performed in order to determine the effective dose of saffron on both cell lines. The cell cycle progress was examined by Flow cytometric analysis. Apoptosis was assessed using Annexin-PI-staining and Western Blotting for caspase 3 and PARP cleavage. Autophagy was determined by Western Blotting of the light chain 3 (LC3)-II and Beclin 1 proteins. The protein content of phospho-H2AX (γH2AX), a sensor of DNA double strand breaks, was also analyzed by Western Blotting.

**Results:**

Saffron extract induced a p53-dependent pattern of cell cycle distribution with a full G2/M stop in HCT116 p53 wildtype cells. However, it induced a remarkable delay in S/G2 phase transit with entry into mitosis in HCT116 p53 −/− cells. The apoptotic Pre-G1 cell fraction as well as Annexin V staining and caspase 3 cleavage showed a more pronounced apoptosis induction in HCT116 p53 wildtype cells. Obviously, the significantly higher DNA-damage, reflected by ɣH2AX protein levels in cells lacking p53, was coped by up-regulation of autophagy. The saffron-induced LC3-II protein level was a remarkable indication of the accumulation of autophagosomes, a response to the cellular stress condition of drug treatment.

**Conclusions:**

This is the first study showing the effect of saffron in HCT116 colorectal cancer cells with different p53 status. Saffron induced DNA-damage and apoptosis in both cell lines. However, autophagy has delayed the induction of apoptosis in HCT116 p53 −/− cells. Considering the fact that most tumors show a functional p53 inactivation, further research is needed to elucidate the long-term effects of saffron in p53 −/− tumors.

## Background

Colorectal cancer is one of the most common malignancies in the world with approximately one million new cases diagnosed every year [1]. The high incidence of the disease and its associated morbidity and mortality has brought colorectal cancer to the center of cancer research, namely its aetiology, diagnosis and treatment. In the last few decades, the acquired knowledge of the molecular biology of the disease and the development of new therapeutic strategies has been steadily increasing [2].

Natural products encompass three main categories of compounds, phenylpropanoids, isoprenoids, and alkaloids, which are widely distributed in plant foods and medicinal herbs [3-5]. This large array of molecules is crucial to human nutrition and health. Plant-derived foodstuffs and beverages also constitute the so-called functional foods and beverages, which include mainly fruits, vegetables, herbs and spices [6]. Saffron, the dried styles (stigmata) of *Crocus sativus* L. flowers, is widely known as a spice and its uses in traditional medicine are well established and date back nearly 3000 years, spanning many continents, civilizations, and cultures [7,8].

Saffron is characterized by its unique aroma, color, and flavor and is cultivated in various parts of the world, particularly in Iran and Spain [9,10]. Cancer chemoprotection is based on the use of exogenous phytochemicals to enhance endogenous mechanisms against various stages of cancer development. There has been a great deal of interest in exploring the chemopreventive properties of herbs and plants. For instance, saffron extracts have the potential to make a major contribution to effective chemopreventive therapy [11].

Studies in animal models and with cultured human malignant cell lines have demonstrated anti-tumor and anti-cancer activities of saffron [12-17]. Different hypotheses for anti-carcinogenic and anti-tumor effects of saffron and its ingredients have been proposed, including (a) inhibition of synthesis of DNA and RNA, but not protein [14], (b) ability to scavenge free radicals [18,19] (c) involvement in the metabolic conversion of carotenoids to retinoids [20], (d) mediation of interactions of carotenoids with topoisomerase II, an enzyme involved in cellular DNA–protein interaction [14], (e) promotion of interactions mediated via lectins [21].

Most recently, we have shown pro-apoptotic and anti-inflammatory effects of saffron extract against hepatocellular carcinoma both in vitro and in vivo [11]. However, the role of the tumor suppressor p53 in saffron-induced programmed cell death has yet to be adequately explored in colorectal cancer. Apoptosis is a gene regulated phenomenon that is important both in physiological and pathological conditions. The regulatory mechanisms of apoptosis include, but are not limited to, caspases and bcl-2 family proteins [22,23]. Since more than 50% of colorectal carcinomas are characterized by a loss of p53 function, here we will analyze the anti-proliferative and pro-apoptotic effects of saffron in HCT colon cancer cell lines with different p53 status.

## Methods

### Plant material and preparation

Stigmata of pure saffron were obtained from the Golpeech Saffron (http://www.golpeechsaffron.com, Register No.: 82558, Mashad, Iran) and voucher sample was preserved for reference in the herbarium of UAEU. Five hundred grams of the stigma materials were extracted at UAEU with 80% (v/v) aqueous ethanol and the mixture was macerated for five days at 4°C. The resulting mixture was then filtered dried under reduced pressure in a rotary evaporator at 40°C to give water and ethanol crude extracts.

### Cell culture and treatment

Human colon cancer HCT116 cell lines (HCT wildtype and HCT p53−/−), obtained from ATCC, Manassas, VA, were maintained in RPMI and DMEM, respectively, and supplemented with 10% fetal bovine serum, penicillin (100 U/ml), and streptomycin (100 μg/ml) inside a humidified incubator with 5% CO_2_ and 95% room air. Cells were subcultured every 4–7 days with trypsin/EDTA (1:250) (PAA Laboratory, Germany). Cells were treated with several concentrations of saffron extract for several time points.

### MTT cell proliferation assay

The 3-(4,5-Dimethylthiazol-2-yl)-2,5-diphenyltetrazolium bromide (MTT), a yellow tetrazole) proliferation assay was utilized in HCT116 cell lines (HCT wildtype and HCT p53−/−) to assess the dose-dependent effect of saffron extract on cell proliferation. Cells (10^4^) were plated and grown in 200 μl of growth medium in 96-well microtiter plates. After an overnight attachment period, cells were treated with varying concentrations of saffron extract (0.25, 0.5, 1, 2, 3, 4, 5 mg/ml) prepared from a 100 mg/ml stock solution dissolved in water for 24 and 48 h. All studies were performed in triplicates and repeated three times independently. Cell growth was quantified by the ability of living cells to reduce the yellow dye, MTT, to a purple formazan product. Cells were incubated with MTT (Sigma-Aldrich, Steinheim, Germany) at 37°C in a humidified 5% CO_2_ atmosphere for 2 h. The MTT formazan product was then dissolved in DMSO, and absorbance was measured at 570 nm in a microplate reader.

### Crystal violet assay

As a second method for determining the cytotoxicity of saffron on HCT116 cells we used the crystal violet assay. Upon solubilization, Cells (7.5 × 10^3^) were seeded and grown in 200 μl of growth medium in 96-well microtiter plates. They were allowed to attach overnight before saffron treatment as described for the MTT assay. After washing with PBS the cells were incubated and were slightly shaked at RT with 50 μl staining solution (0.5% crystal violet, 20% methanol) which stains DNA. The plate was washed 2 times with dH_2_O and dried completely. The uptaken crystal violet was solubilized by addition of 200 μl of methanol and 15 min incubation on a shaker. Finally, the amount of dye taken up by the monolayer was quantified by measuring the absorbance at 570 nm in a microplate reader. All studies were performed in triplicates and repeated three times independently.

### Flow cytometric analysis of DNA content

One day before treatment, cells were seeded at a density of 1.2 × 10^6^ cells per well of a 6-well plate. After the indicated times, the cells were harvested by trypsin release, washed twice with phosphate buffered saline (PBS) resuspended in 0.5 ml icecold PBS and fixed over night with 4 ml ice cold 70% ethanol. Low molecular weight DNA fragments were extracted by 10 min incubation with extraction buffer (9 parts 50 mM Na_2_HPO_4_, 1 part 25 mM citric acid and 0.1% triton x-100; pH 7.8). After centrifugation the cells were resuspended and incubated in the dark at RT in 0.4 ml staining buffer (10 mM PIPES, 100 mM NaCl, 2 mM MgCl_2_, 0.1% triton x-100; pH 6.8) with the addition of 250 μg RNase and propidium iodide (50 μg/ml final concentration). Distribution of cell cycle phases with different DNA contents was determined using a flow cytometer FACS CantoII (Becton-Dickinson, CA, USA). Sub G_1_ cells in flow cytometric histograms were considered apoptotic cells. Analysis of cell cycle distribution and the percentage of cells in the G_1_, S, and G_2_/M phases of the cell cycle were determined using the cell cycle platform of the FlowJo software with the Watson pragmatic model (Tree Star, Ashland OR, USA).

### Quantification of Apoptosis by AnnexinV-PI staining

To detect apoptosis, the AnnexinV-FLUOS kit (Roche Diagnostics, USA) was used. Cells were treated for 24 and 48 h with saffron extract. After washing twice in PBS, 1 × 10^6^ cells were stained with 100 μl annexin V staining solution, consisting of 20 μl FITC-conjugated annexin V reagent (20 μg/ml), 20 μl isotonic propidium iodide (50 μg/ml), and 1000 μl of 1 M/L HEPES buffer, for 15 minutes at room temperature. Cells were analyzed on a flow cytometer FACS CantoII (Becton-Dickinson, CA, USA) using a 488 nm excitation and 530/30 nm band pass filter for fluorescein detection and a long pass filter 2P670 nm for propidium iodide detection after electronic compensation. Since positive annexin V staining indicates apoptotic and necrotic cells, propidium iodide-positive cells were used to measure late apoptotic cells and necrotic cells whereas annexin V-positive and propidium iodide-negative cells were counted as early apoptotic cells.

### Western Blotting

Whole cell lysates were prepared from both HCT116 cell lines tumor cells. Protein concentration of lysates was determined with Bio-Rad Dc Protein Assay (BioRad Laboratories, Hercules, CA, USA), and 30 μg proteins were loaded onto 12% SDS-polyacrylamide gel electrophoresis (voltage: 30 mA, running time 1.5-2 h). Gels were transferred to nitrocellulose membranes before immunodetection processing with antibodies against phospho-H2AX (Millipore), caspase 3, phospho-H3, PARP (all 3 Cell Signaling Technology Inc., MA, USA), LC3 (Nanotools, Germany), H3 (Actife Motif, CA, USA), cyclin B1 (Santa Cruz, CA, USA) and with secondary antibodies (anti-mouse or anti-rabbit IgG peroxidase conjugated; Pierce, Rockford, IL, USA). Bound antibodies were detected by incubating the blots in West Pico chemiluminescent substrate (Pierce, Rockford, IL, USA). The level of immunoreactivity was measured as peak intensity using an image capture and analysis system (GeneGnome, Syngene, UK). Hybridization with anti-GAPDH was used to control equal loading and protein quality.

### Statistical analysis

Data were analyzed using the Student *t*-test to calculate the significance values; a probability value (p) <0.05 was considered statistically significant.

## Results

### Saffron has an anti-proliferative effect in p53 isogenic HCT116 cell lines

**Figure 1:**
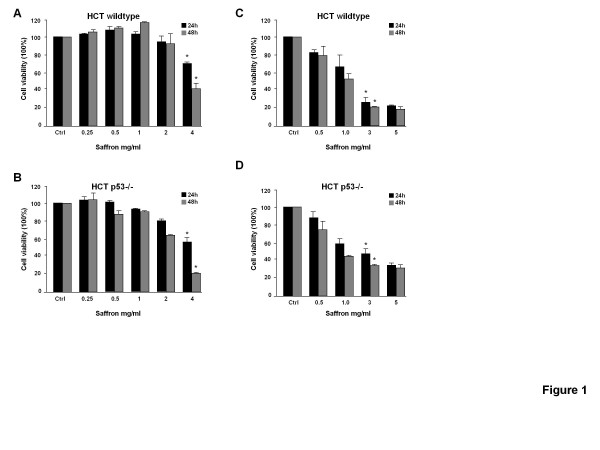
**Anti-proliferative and cytotoxic effects of saffron in HCT116 p53 wildtype and HCT116 p53 −/− cells.**MTT viability test showing the HCT116 p53 wildtype and HCT116 p53 −/− cells untreated (ctrl) or treated with different concentrations of saffron (0.25 to 4.0 mg/ml) for 24 and 48 h (**A** and **B**). The MTT data shown are performed in triplicates. Cell numbers were measured using the crystal violet technique after saffron treatment for 24 and 48 h (**C** and **D**). Results are means ± s.d. (n = 3). (*, Saffron treatment was statistically significant against the control for P < 0.05). (#, Saffron treatment was statistically significant in HCT116 p53 wildtype against HCT116 p53 −/− cells for P < 0.05).

To determine the anti-viability effect of saffron, both cell lines were treated with various concentrations of saffron for 24 and 48 h. MTT (3-(4,5-Dimethylthiazol-2-yl)-2,5-diphenyltetrazolium bromide, a yellow tetrazole) is reduced to purple formazan in living cells and using the MTT test we can measure the viability and proliferation of cells after drug administration. Saffron reduced the proliferation of HCT116 cells in a time- and concentration-dependent manner. The most remarkable effects were observed at concentrations between 2 and 4 mg/ml (Figure 1A and B). The mitochondrial function of HCT116 p53−/− cells was more efficiently diminished by the drug at doses between 1 and 4 mg/ml.

Crystal violet staining was used to determine the number of viable cells after drug exposure. It confirmed that saffron significantly decreased the viability of both cell lines (p < 0.05) at 3 mg/ml (Figure 1C and D). HCT116 p53 wildtype cells died significantly more than HCT116 p53−/− cells with 3 and 5 mg/ml at both time points, 24 h (26% versus 40% respectively) and at 48 h (14% versus 28%, respectively) (p < 0.05).

### Saffron induces a p53-dependent cell cycle arrest in HCT116 cells

**Figure 2:**
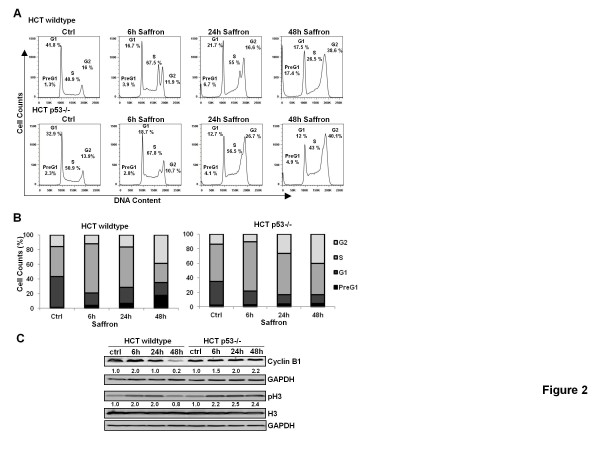
**Saffron induced cell cycle arrest in HCT116 cells.**(**A**) Two cell types, HCT116 p53 wildtype and HCT116 p53 −/− cells, untreated (Ctrl) or treated with 3 mg/ml saffron for 24 and 48 h, were harvested and DNA was stained with PI for flow cytometric analysis of DNA content with FACScan flow cytometry. (**B**) Quantitative analysis of percentage gated cells at PreG_1_, G_1,_ S and G_2_ phases in the HCT116 p53 wildtype and HCT116 p53 −/− cells. Results are means ± s.d. (n = 3). (**C**) Western Blot analysis for Cyclin B1, Histone 3, and phosphoHistone 3 (pH3) expressions in HCT116 p53 wildtype and HCT116 p53 −/− cells, untreated (Ctrl) or treated with 3 mg/ml saffron for 6, 24, and 48 h. (*, Saffron treatment was statistically significant against the control for P < 0.05).

To examine the effect of saffron on cell cycle dynamics, flow cytometric analysis was performed. Figure 2A and B showed that saffron induced a transient S phase arrest at 6 h in both cell lines and there were p53-dependent effects on cell cycle phase distribution at later time points. HCT116 p53 wildtype cells still showed a pronounced S-phase arrest at 24 h. The HCT116 p53−/− cells, however, resumed proliferation and released cells into G2/M phase without showing a remarkable increase in Pre-G1 apoptotic cell fraction. Interestingly, HCT116 p53 wildtype cells showed a delay in S/G2 checkpoint passage returning to proliferation later at 48 h. That was clearly shown by a significant decrease (p < 0.05) in S phase and an increase in G2 phase. In contrast to HCT116 p53 −/− cells 17.4% of cells were simultaneously released from the cell cycle. This arrest of proliferation-execution process was reflected by a double S-phase peak in both cell lines but was more obvious in HCT116 p53 wildtype cells (Figure 2A and B).

Through analyzing two key regulators of G2/M arrest, namely cyclin B1 and phospho-Histone 3 (pH3), we confirmed the FACS analysis. Western blot analysis showed that there was an early 2-fold up-regulation of cyclin B1 in HCT116 p53 wildtype cells followed by a decrease to the control level at 24 h. Also the cyclin B1 protein has nearly completely disappeared at 48 h after saffron treatment (Figure 2C). As expected, this decrease was associated with a down regulation of mitosis-specific pH3 suggesting no M phase transition (Figure 2C).

On the other hand, HCT116 p53−/− cells showed a continuous increase in cyclin B1 protein levels with time and finally failed to decrease the levels of cyclin B1 and pH3 at 48 h. This suggests that saffron treatment causes HCT116 p53 −/− cells to enter mitosis and undergo cell death possibly at later time points (Figure 2C).

### Saffron induces p53-dependent caspase 3 activation in HCT116 cells

**Figure 3:**
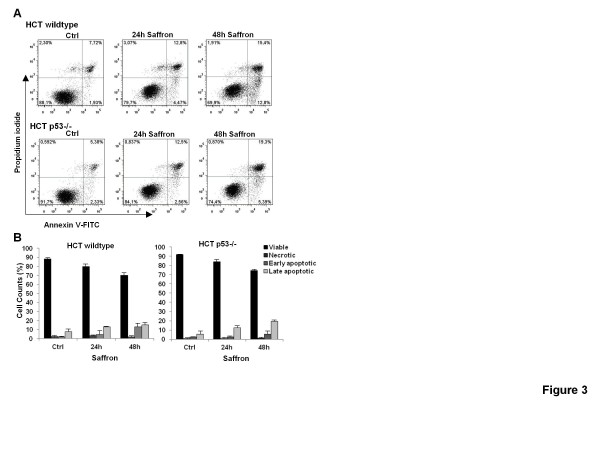
**Pro-apoptotic effects of saffron.**(**A**) Annexin-PI measurements of untreated cells (Ctrl) and the two cell types, HCT116 p53 wildtype and HCT116 p53 −/− cells, treated with 3 mg/ml saffron for 24 and 48 h. The profile represents Annexin-V-FITC staining in *x* axis and PI in *y* axis. (**B**) Quantitative analysis of percentage gated for viable, necrotic, early apoptotic, and late apoptotic HCT116 p53 wildtype and HCT116 p53 −/− cells treated with 3 mg/ml saffron for 24 and 48 h. Results are means ± s.d. (n = 3). (*, Saffron treatment was statistically significant in HCT116 p53 wildtype against HCT116 p53 −/− cells for P < 0.05).

**Figure 4:**
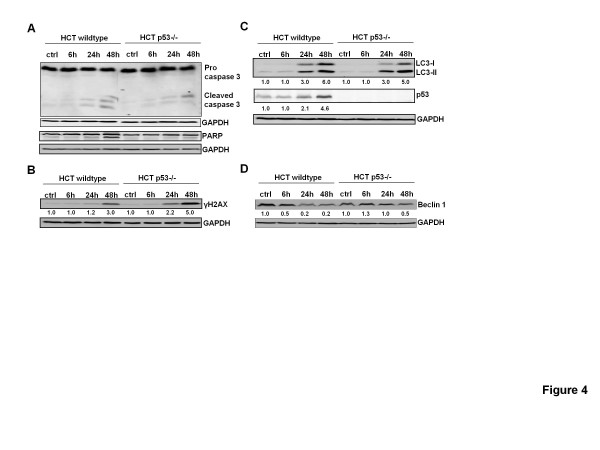
**Saffron induced DNA damage and autophagy.**(**A**) Western Blot analysis for caspase 3 and PARP cleavage in two cell types, HCT116 p53wildtype and HCT116 p53 −/− cells, untreated (Ctrl) or treated with 3 mg/ml saffron for 6, 24, and 48 h. (**B**) Western Blot analysis for γH2AX expression in HCT116 p53 wildtype and HCT116 p53 −/− cells, untreated (Ctrl) or treated with 3 mg/ml saffron for 6, 24, and 48 h. (**C**) Western Blot analysis for LC3 expression in HCT116 p53 wildtype and HCT116 p53 −/− cells, untreated (Ctrl) or treated with 3 mg/ml saffron for 6, 24, and 48 h. (**D**) Western Blot analysis for Beclin 1 expression in HCT116 p53 wildtype and HCT116 p53 −/− cells, untreated (Ctrl) or treated with 3 mg/ml saffron for 6, 24, and 48 h.

Next we confirmed apoptosis induction in the two HCT116 p53 isogenic cell lines using the Annexin-PI staining. We showed a similar time-dependent increase in the number of apoptotic cells after saffron treatment in both cell lines compared to the basal apoptosis levels (Figure 3A and B). However, the early apoptotic fraction (Annexin-positive and PI-negative) was significantly higher in HCT116 wildtype cells (p < 0.05) at 48 h (12.8% versus 5.4%, respectively). Examining the caspase activation, we indicated a higher increase of the cleaved caspase 3 products in HCT116 p53 wildtype cells. This was accompanied by an increase in cleavage of its target PARP (Figure 4A) showing more apoptosis and suggesting that this saffron-induced DNA-damage is mediated through the gate keeper p53. Indeed, there was an up-regulation of p53 protein reaching 4.6-fold values at 48 h. In parallel, there was a remarkable up-regulation in DNA damage after saffron treatment as reflected by an increase in protein levels of γH2AX, a sensor of DNA double strand breaks (Figure 4B). But HCT116 p53 −/− cells showed an increase in γH2AX already after 24 h and the levels where approximately 2.5-fold higher compared to the HCT116 p53 wildtype cells at 48 h (Figure 4B).

### Saffron induces a massive autophagic cell death in HCT116 p53 −/− cells

We next ask the question, what is protecting HCT116 p53 −/− cells from apoptosis after the observed severe DNA damage. In order to address this, we investigated whether saffron induces hallmarks of autophagy in a p53-dependent manner. The level of LC3-II, a protein that accumulates as a result of increased autophagosome formation [24], was assessed. Indeed, saffron induced a more pronounced conversion from LC3-I to LC3-II in HCT116 p53−/− cells than in HCT116 p53 wildtype cells after 24 h and 48 h, (Figure 4B), although there was a higher basal rate of autophagy in HCT116 p53−/− cells. Studying Beclin 1, which is known to interfere with autophagic vesicle formation, allowed us to find a higher beclin 1 protein levels in the HCT116 p53 −/− cells after saffron treatment. We, therefore, suggest that autophagic disposal of damaged cell debris might reduce or delay the cell death induction after saffron treatment in HCT116 p53 −/− cells.

## Discussion

Natural products have long been used to prevent and treat diseases including cancers and might be good candidates for the development of anti-cancer drugs [25]. Saffron, a commonly-used spice and food additive, is known for its anti-cancer and anti-tumor properties [12-16].

Because most of the in vivo studies were interested in the isolated bioactive compounds of saffron, little research has been done to examine anti-cancer properties of saffron in its natural form. Tavakkol-Afshari and colleagues [26] reported that 96% ethanol saffron extract is selectively cytotoxic against epithelial-like human hepatocellular carcinoma cells (HepG-2) as well as human cervical carcinoma cells (HeLa) but nontoxic towards normal mouse fibroblast cells (L929). The additive and synergistic effects among the different phytochemicals in saffron however, may enhance its anti-carcinogenic properties [27]. Based on those reports, more studies are necessary to determine the beneficial effects of saffron in its natural form using human subjects [9].

In this study, we analyzed the possible role of p53 in the cytotoxic, anti-proliferative and pro-apoptotic effects of saffron on two p53 isogenic HCT116 cell lines. Cell proliferation inhibition test using MTT viability assay as well as crystal violet staining confirmed that ethanolic extract of saffron has cytotoxic activity against HCT116 cells. The present results were in agreement with previous reports indicating that saffron and its ingredients possessed anti-tumor and anti-cancerogenic activities and have no cytotoxic effect on non-malignant cells [9,26,27]. Consistent with our previous study on HepG-2 cells [11], saffron extract induced a significant toxicity in HCT116 after 48 h. Similarly, HCT116 cells were shown to have a significant higher sensitivity to saffron extract compared to other colorectal cancer cell lines; SW-480 and HT-29 [27]. The observed apoptotic induction in HCT116 p53 wildtype cells obviously resulted from DNA damage as reflected by the up-regulation of the double-stranded DNA breakage marker, γH2AX, suggesting an additional role of saffron in sensitizing cancer cells to the effects of other chemotherapeutics.

In cancer chemotherapy, cancer programmed cell death has been emphasized and shown to be mediated by many factors. The tumor suppressor p53 mediates apoptosis, cell cycle regulation and DNA repair in response to a wide range of cellular stresses. Mutations in p53 have been found in almost 50% of all colorectal cancers worldwide [28] and at high frequencies in many different cancers [2]. We investigated whether saffron treatment induced p53-dependent cell cycle arrest, apoptosis or autophagy in HCT116 cell lines. Sluggish S-phase transit was a remarkable effect of saffron in both cell lines. To better understand the subsequent G2/M arrest, we studied the Cyclin B1 protein levels because a proper Cyclin B1 regulation is known to be necessary for the entry into mitosis [29]. The low Cyclin B1 and pH3 levels in HCT116 p53 wildtype cells reflect that saffron induces a full G2 arrest after 48 h of treatment without allowing the cells to enter the M phase. Cells without p53 but high Cyclin B1 and pH3 levels clearly enter the M phase and further accumulate severe saffron-induced DNA damage as reflected by the increase in pH2AX protein levels.

The role of p53 in autophagy, programmed cell death form II, remains controversial with studies suggesting presence of p53, as well as absence of p53, as inducer of autophagy [30]. In the present study, both cell lines showed accumulation of autophagosomes after saffron treatment monitored by the increase in the lipidated LC3-II protein. There are two forms of LC3 proteins: the cytoplasmic LC3-I and the autophagosome membrane-bound LC3-II form. The failure in repairing the massively damaged DNA by a lack in p53 led to a significant higher LC3-II proportion after saffron treatment. This effect has been observed also after prolonged starvation [31] and has been interpreted as an increased functional autophagy in p53 −/− cells. In the absence of p53, autophagosome recycling is possibly less effective and the observed increase in LC3-II forms after saffron treatment of HCT116 p53 −/− cells could also reflect the aberrant accumulation of static autophagosomes. This would slow down the autophagic flux, cause accumulation of aberrant protein intermediates and would have a later effect on apoptosis. Thus autophagy would ultimately lead to a delay in apoptosis and protection of cells from cell death. The time interval observed in our study suggests a rather pro-survival role of saffron-induced autophagy. Indeed, the role of autophagy in cancer development is complex, as it has been implicated in both tumor survival and tumor cell death [32,33]. At the present time, we still do not know the genes responsible for inducing autophagy in the p53 −/− cells after saffron treatment. We could however speculate that a p53 homolog such as p73 may have been activated in p53−/− cells and modulated autophagy [34] or mild apoptosis. In this regard, it has been shown that p73 interacts with p53-responsive elements and induces transcription of p53-inducible genes [35].

Despite the accumulating evidence demonstrating that saffron may be a promising cancer therapy agent, mechanisms of saffron anti-cancer actions are still largely unknown. Many proposed mechanisms have been reported. Bathaie and colleagues [36] found that saffron carotenoids directly bind to DNA minor grooves and induce conformational changes of targeted DNA. The induction of apoptosis by saffron has been reported to play an essential role in the death of human hepatocellular carcinoma cells and HeLa cells [11,26]. Although saffron demonstrates potential as an anti-cancer drug, further research is needed to elucidate the mechanisms and effects of saffron in p53 −/− tumors. The therapeutic endpoint in p53 −/− tumor cells has to be clarified using long term assays and in vivo models.

## Conclusion

In conclusion, data from this study suggests that saffron shows a p53-dependent efficacy in colorectal cancer. There seems to be a pro-survival role of autophagy in saffron-induced damage response in p53 −/− tumors. Considering the popularity of herbal use in cancer patients, saffron and especially its active ingredients should be investigated further as a viable option in the treatment of colorectal cancer.

## Competing interests

The authors declare that they have no competing interests.

## Authors’ contributions

RSS designed the study and supervised the experimental work. She helped with interpreting the data and writing the manuscript. KB wrote the manuscript and designed the Figures. KB and SD have done the cell culture experiments and Western Blot analyses. AA supplied the saffron extract and other reagents and was involved in drafting the manuscript and has reviewed the final version prior publication. JSL performed the FACS PI and Annexin-PI analysis and prepared the corresponding figures. All authors read and approved the final manuscript.

## Pre-publication history

The pre-publication history for this paper can be accessed here:

http://www.biomedcentral.com/1472-6882/12/69/prepub
